# Kinesio taping in knee osteoarthritis: mechanisms, clinical evidence, and rehabilitation implementations

**DOI:** 10.1007/s00296-026-06209-x

**Published:** 2026-06-22

**Authors:** Kubra Ayse Dasdemir, Nihan Karatas

**Affiliations:** 1 Department of Health Care Services, Dogubayazit Ahmedi Hani Vocational School, Agri Ibrahim Cecen University, Agri, Turkey; 2 Department of Physiotherapy and Rehabilitation, Faculty of Health Sciences, Gazi University, Ankara, Turkey

**Keywords:** Osteoarthritis, knee, Kinesio tape, Pain management, Muscle strength, Proprioception, Range of motion, articular

## Abstract

Kinesio taping is widely used as a non-pharmacological adjunct in knee osteoarthritis (OA), although its mechanisms and clinical value remain debated. This narrative review synthesizes current evidence on kinesio taping in knee OA, addressing mechanisms and effects on pain, function, muscle activation, proprioception, and range of motion (ROM). PubMed/MEDLINE, Scopus, Web of Science, Directory of Open Access Journals (DOAJ), and Google Scholar were searched through 19 May 2026 using MeSH terms “Osteoarthritis, Knee” and “Athletic Tape” with related keywords. Eligible sources included systematic reviews, meta-analyses, randomized controlled trials, observational studies, and appraised qualitatively with interpretive weight ranked by study design. Kinesio taping may provide short-term pain reductions that fall below minimal clinically important difference (MCID) (15–20 mm VAS). Functional gains are modest and inconsistent, mostly below WOMAC MCID (17%). Although kinesio taping does not increase muscle strength, it may modulate quadriceps-hamstring coactivation and neuromuscular control during low-load tasks. Proprioceptive gains are reportedly angle-specific (significant at 15° and 30°). Flexion ROM increases modestly, whereas extension changes little. Compared with other adjunctive treatments, it shows lower or comparable effects. Adverse effects are confined to minor, self-limiting skin reactions. Current evidence does not support the routine use of kinesio taping as a standalone treatment for knee OA. Kinesio taping may be considered as a short-term adjunct to exercise and education in selected patients, as it may facilitate symptom relief, sensory feedback, and low-load activity. Methodological heterogeneity remains a significant limitation, and randomized controlled trials are needed.

## Introduction

Osteoarthritis (OA) is the most common musculoskeletal disease on a global scale [1, 2]. The knee is the most frequently affected joint because of its weight-bearing and repetitive motion [3]. The burden of knee OA is expected to increase substantially by 2050 [4]. Given the current lack of treatments to halt disease progression or reverse joint degeneration, therapeutic approaches primarily focus on reducing symptoms and improving functional capacity [5]. International guidelines recommend exercise, weight management, and patient education as the core non-pharmacological interventions for knee OA, and other modalities are used as adjuncts to these or to improve adherence [6].

Kinesio^®^ tape is a 100% cotton, latex-free, body-heat-activated tape with similar thickness and elasticity to skin [7]. Although frequently preferred in individuals with knee OA, there is no clear consensus in the literature regarding its clinical effectiveness. Several randomized controlled trials and meta-analyses have demonstrated that Kinesio^®^ taping is superior to sham taping for pain, range of motion, and functional performance [8, 9], whereas other studies have found no additional benefit over sham taping or standard care for pain, edema, or muscle strength [10–12]. These conflicting results stem from significant differences in factors such as the techniques used, tension percentage, tape direction, implementation duration, and follow-up frequency [13].

A recent systematic review and meta-analysis concluded that Kinesio^®^ taping may reduce pain and improve function and flexion range of motion in the short term, even when conventional physiotherapy is not applied. However, its long-term effects remain unclear, and its integration with broader rehabilitation strategies has not been examined in depth [14]. Consequently, existing studies have primarily focused on pooled effect estimates, offering only a limited synthesis of the proposed neurophysiological and biomechanical mechanisms, as well as the interaction of Kinesio^®^ taping with task-specific responses and basic rehabilitation strategies. To address this gap, this narrative review aims to provide an overview of the effects of Kinesio^®^ taping on pain, function, muscle performance, proprioception, and range of motion in knee OA, and to discuss these findings in the context of proposed mechanisms and their cautious integration with rehabilitation strategies.

## Method

The study had a narrative review design examining the clinical efficacy, mechanisms of action, and outcomes of Kinesio^®^ taping in individuals with knee OA.

### Search strategy

The search strategy was designed and reported in accordance with established recommendations for comprehensive literature searches in narrative reviews [15]. The search was conducted in PubMed/MEDLINE, Scopus, Web of Science, Directory of Open Access Journals (DOAJ), and Google Scholar. There were no restrictions on the start date of the searches, and the literature search covered studies up to 19 May 2026. The search strategy was developed according to the features of each database. The search strategy also includes Medical Subject Headings (MeSH) terms from the National Library of Medicine: “Osteoarthritis, Knee”[MeSH Terms], “Athletic Tape”[MeSH Terms]. Search conditions were combined with the Boolean operator “AND” in each database (Table 1).

**Table 1:** Search strategy

Database	Search condition	
	Knee osteoarthritis	Kinesio tape
PubMed/MEDLINE [Title/Abstract]	“osteoarthritis, knee” [MeSH Terms] OR “osteoarth* knee” OR “knee osteoarth*” OR “osteoarthritis of knee” OR “osteoarthritis of the knee” OR “knee OA” OR KOA OR gonarth*	“Athletic Tape”[MeSH Terms] OR “kinesio tap*” OR “kinesiology tap*” OR kinesiotap* OR “elastic tap*” OR “neuromuscular tap*” OR “therapeutic tap*” OR “Kinesio Tex”
Scopus [Title/Abstract/Keywords]	“osteoarth* knee” OR “knee osteoarth*” OR “osteoarthritis of knee” OR “osteoarthritis of the knee” OR “knee OA” OR KOA OR gonarth*	“kinesio tap*” OR “kinesiology tap*” OR kinesiotap* OR “elastic tap*” OR “neuromuscular tap*” OR “therapeutic tap*” OR “Kinesio Tex”
Web of Science [Topic]	“osteoarth* knee” OR “knee osteoarth*” OR “osteoarthritis of knee” OR “osteoarthritis of the knee” OR “knee OA” OR KOA OR gonarth*	“kinesio tap*” OR “kinesiology tap*” OR kinesiotap* OR “elastic tap*” OR “neuromuscular tap*” OR “therapeutic tap*” OR “Kinesio Tex”
DOAJ [All fields]	“knee osteoarthritis” OR “osteoarthritis of the knee” OR “osteoarthritis of knee” OR “knee OA” OR KOA OR gonarthrosis OR gonarthritis	“kinesio taping” OR “kinesio tape” OR “kinesiology taping” OR “kinesiology tape” OR kinesiotaping OR kinesiotape OR “elastic taping” OR “elastic tape” OR “neuromuscular taping” OR “neuromuscular tape” OR “therapeutic taping” OR “therapeutic tape” OR “Kinesio Tex”
Google Scholar [All fields]	“knee osteoarthritis” OR “osteoarthritis of the knee” OR “osteoarthritis of knee” OR “knee OA” OR KOA OR gonarthrosis OR gonarthritis	“kinesio taping” OR “kinesio tape” OR “kinesiology taping” OR “kinesiology tape” OR kinesiotaping OR kinesiotape OR “elastic taping” OR “elastic tape” OR “neuromuscular taping” OR “neuromuscular tape” OR “therapeutic taping” OR “therapeutic tape” OR “Kinesio Tex”

*DOAJ* Directory of Open Access Journals

### Study selection

The inclusion criteria are as follows; (1) included adult participants (≥ 18 years) with a clinical or radiographic diagnosis of knee OA; (2) evaluated Kinesio^®^ taping as the intervention, either alone or as an adjunct to other conservative treatments; (3) reported at least one outcome related to pain, physical function, muscle activity or strength, proprioception, or knee range of motion; (4) were systematic reviews, meta-analyses and peer-reviewed studies (randomized controlled trials, quasi-experimental or observational studies). Studies for which the full text could not be accessed (e.g., abstracts, conference papers), studies not published in peer-reviewed journals, and study protocols were not included.

No language restrictions were applied during the search. By scanning the titles and abstracts, irrelevant studies were eliminated, and the full texts of the remaining studies were examined. Also, the reference lists of the selected studies were reviewed. The evidence was appraised qualitatively. Study design determined interpretive weight: systematic reviews and meta-analyses ranked highest, followed by randomized controlled trials, and then non-randomized studies. Sample size, methodological transparency, and cross-study consistency were factored in as secondary criteria. Where evidence was conflicting or limited, this was stated explicitly in the narrative synthesis.

## Clinical implementations of kinesio taping in knee osteoarthritis

Kinesio^®^ taping was applied to individuals with knee OA using different techniques, tension levels, and durations to reduce pain, increase joint stability, regulate muscle function, and control edema [16, 17]. Table 2 presents an evidence-informed framework summarizing specific taping parameters alongside necessary adjunctive therapies. Studies generally used “Y”, “I”, and “Fan” cut techniques. Knee OA is not just a cartilage disorder; it is a complex condition that involves edema, muscle weakness, instability, and pain. For this reason, since a single taping technique may not be sufficient for every patient, several studies have combined multiple techniques [18]. The amount of tension applied to the skin varies in studies depending on the desired effect. Tension levels ranged from 15% to 75% for muscle facilitation [19], 75–100% for mechanical correction and ligament support, and 0–15% for lymphatic drainage and pain relief [20]. The wide range of tension levels in the literature, even for specific purposes, leaves it unclear what amount of tension exceeds the therapeutic threshold. The goal of muscle facilitation is generally to support and activate the weakened quadriceps muscle [7]. The most common method is the implementation of a Y-shaped strip from the origin to the insertion [14]. An I-shaped strip was used to correct patella movement within the femoral groove, stabilize the patella, and reduce mechanical stress on the joint [11, 16, 21]. The fan-shaped strip tape is often used to reduce joint effusion [16, 22]. In the literature, it is debatable whether the direction of implementation of the taping (from origin to insertion or from insertion to origin) actually creates the desired facilitation or inhibition effect on muscle activation [23, 24]. Some studies argued that the sensory input generated, rather than the direction, is the primary factor [25, 26]. Although there is no gold standard regarding the duration and frequency of implementation, the common clinical approach is to renew the tape 1–2 times a week, leave it on the skin for 3–4 days, and continue treatment for 4 to 6 weeks [8, 18]. Knee OA severity generally ranges from stages 1 to 4 according to the Kellgren-Lawrence (KL) classification, with most studies focusing on stages 2 and 3 (mild-moderate) and limits the generalization of results to stage 4 (advanced) individuals [19, 27]. Most studies have compared Kinesio^®^ taping with sham taping or Kinesio^®^ tape and exercise in combination with exercise alone or conventional treatment alone [8, 14, 18, 28]. Although its implementation with other treatments makes it difficult to isolate the individual effect of Kinesio^®^ taping, it reflects the multimodal treatment approach frequently used in clinical practice.

**Table 2:** Clinical application framework

Patient subgroup (KL grade)	Clinical goal	Tape technique	Tension	Duration	Suggested adjuncts	References
KL I-III Pain-dominant / activity pain	Short-term analgesia; reduction of activity- and walking-related pain	Y-strip over rectus femoris/quadriceps; I-strip over rectus femoris with Y-strip proximal to superior patellar border; optional I-strip across patella for mechanical correction	15–25% (Y/I sensory, paper-off ends) 25–50% (facilitation) 75–100% (corrective patellar I-strip only)	2–6 weeks (2 applications/week, 3–4 days per application)	Exercise; patient education; weight management	[8, 9, 11, 14, 16, 18–21, 27, 34–36, 40]
KL I-III Edema-dominant / post-flare swelling	Lymphatic drainage; reduce peri-articular edema; Secondary analgesia reduce	Fan-cut or web drainage strip directed from proximal collector lymph nodes toward the knee; peri-patellar lifting/decompression strips	0–15% (paper-off / minimal tension) to maximise convolution and lifting effect	3–4 days per application; symptom-driven (typically 1–3 applications during the active edema phase).	Exercise; education; weight management; activity modification; rest; cryotherapy	[10, 11, 16, 20, 64]
KL I-III Quadriceps inhibition / weakness	Neuromuscular facilitation of the quadriceps; restoration of agonist–antagonist (quadriceps–hamstring) balance; reduction of hamstring co-activation	Y-strip on rectus femoris/quadriceps for facilitation; Y-strip on hamstrings	25–50% (facilitation); some protocols 50–75%	3–6 weeks (2–3 applications/week, 3–4 day per aplication)	Quadriceps and hip-abductor strengthening; combined open/closed kinetic-chain exercise; gait and balance training; education; weight management	[16, 19, 21, 23, 34, 35, 46, 49, 50]
KL I-III Proprioceptive deficit / impaired balance	Increase cutaneous mechanoreceptor input; improve joint position sense, postural control and walking confidence, particularly at near-extension angles	Y-strip over quadriceps (sensory facilitation) with light tension; peri-patellar Y/I or mechanical-stabilization pattern around the knee; bilateral application may be considered when proprioception is the primary target	10–25% (sensory/stabilization)	4–6 weeks	Balance training; tai-chi or neuromuscular exercise; functional gait training; education; weight management; knee brace when instability meaningfully limits ambulation	[9, 17, 20, 36, 42, 53, 54]
KL I-III Mixed pain, edema and functional limitation	Multimodal symptom relief; address pain, peri-articular swelling and quadriceps function within a single treatment plan	Multi-layer combined application: Y-strip over quadriceps (facilitation) + I- or Y-strip across patella (patellofemoral joint unloading) + fan-cut drainage strip. Component selection guided by the dominant symptom; high-tension strips should be avoided over edematous skin	10–25% as a baseline; reserve 50–75% only for the corrective component when patellar loading or maltracking is targeted	2–6 weeks (2–3 applications/week, 3–4 days per application)	Exercise; education; weight management; knee brace if instability dominates	[8, 10, 14, 16, 18–20, 27, 43]
KL IV Advanced structural disease / joint-replacement candidate	Generally not recommended as routine symptomatic management	No standardized routine kinesio taping protocol; individualized, low-risk, low-tension application may be trialled for short-term symptom modulation	Low-to-moderate (10–25%)	If used, restrict to short, symptom-driven cycles (e.g., 1–2 weeks); discontinue if no meaningful benefit	Exercise as tolerated; patient education; weight management	[20– 22, 32, 33]

*KL* Kellgren–Lawrence

## Clinical evidence of kinesio taping in knee osteoarthritis

### Pain outcomes

Pain usually begins insidiously and progresses over time in knee OA. Initially, it is intermittent and activity-related, but in later stages, it develops into persistent pain that continues even at rest and at night. Pain begins with the activation of peripheral nociceptors by mechanical or chemical stimuli, but the process becomes more complex with the effect of central sensitization [29]. The effect of Kinesio^®^ taping on pain is based on various theories. The mechanisms for this analgesic effect are gate control theory hypotheses, resulting from the expansion of the interstitial space by lifting the skin, increased blood and lymph circulation, reduced pressure on nociceptors, and stimulation of mechanoreceptors [7, 30]. An MRI-based analysis have suggested that Kinesio^®^ tape causes tissue deformations involving not only superficial tissues but also deep fascial tissues and muscles, and that this mechanical loading can alter myofascial force transmission throughout the entire extremity. This finding raises the possibility that the analgesic effect of the tape is not limited to skin lifting but may also modulate pain through the deep mechanical effect it creates on fascial and muscle tissues [31]. However, this finding has not been consistently validated by physiological research. Studies have failed to demonstrate reproducible changes in measurable physiological responses [25, 26].

Although the abovementioned mechanisms provide some basis, the clinical significance of the Kinesio^®^ taping effect on pain in knee OA is not clear yet. Some meta-analyses suggest statistically significant short-term pain reductions in favour of Kinesio^®^ taping compared with sham or no taping [8, 14], whereas other meta-analyses conclude that current evidence does not provide strong support for its use for pain improvement in knee OA [20, 32, 33]. At the level of randomized controlled trials, the evidence is similarly inconsistent. While some trials describe short-term reductions in pain in favour of Kinesio^®^ taping [9, 34, 35] others have found no superiority over sham or no taping for either short- or long-term pain control [10, 11, 20]. Even when statistically significant reductions are observed, the magnitude of pain reduction often remains below the minimum clinically important difference (MCID) of 15–20 mm on a 0–100 mm visual analogue scale (VAS), indicating that the observed effects may not lead to a perceptible benefit at the patient level [36]. Similar levels of pain reduction have been reported in sham taping groups in various studies. This has led to debates over whether the observed effect is a genuine physiological response or a placebo effect related to the intervention’s visibility and the emotional and opioid-mediated components of tactile contact [32, 33, 37].

Given the modest and short-term effects observed, Kinesio^®^ taping should be considered an adjunt rather than a standalone intervention for knee OA pain, and is best implemented alongside established treatments such as exercise and education. Evidence suggests that incorporating Kinesio^®^ taping into exercise or physical therapy may yield slightly greater short-term pain relief than those treatments alone, but these gains typically do not reach clinical significance and are confined to the initial follow-up period [18, 38]. Furthermore, any incremental benefit appears limited to early-stage (1–2) knee OA, with no additional effect observed in more advanced-stages (3–4) [21].

In conclusion, current evidence suggests that the analgesic effect of Kinesio^®^ taping in knee OA is modest, short-lasting, and often does not reach clinical significance, especially in advanced stages or when used alone. Kinesio^®^ tape may be used as a low-risk, short-term symptomatic adjuvant in selected individuals with mild-to-moderate knee OA, as part of multimodal rehabilitation programs. However, there is limited current evidence to support its routine usage as a standalone analgesic strategy.

### Functional outcomes

In knee OA, functional limitations, pain, and joint stiffness are significant symptoms, affecting quality of life. Functional limitations typically manifest as impairment in daily living activities such as walking, climbing stairs, squatting, and sitting down and standing up from a chair. As knee OA progresses, decreased quadriceps muscle strength and impaired proprioceptive sensation lead to joint instability, further reducing functional capacity [39].

The effects of Kinesio^®^ taping on function are based on biomechanical and neurophysiological principles. The tape can increase proprioceptive input by stimulating skin receptors, regulate the activation of muscles around the joint, and provide mechanical support [28]. It is suggested that the ability of the tape to lift the skin increases blood and lymphatic circulation, reduces edema, and improves joint range of motion and, therefore, functioning [8]. Also, pain reduction might break the pain-weakness-pain cycle, allowing patients to overcome kinesiophobia and perform functional tasks more comfortably [40].

Several systematic reviews and meta-analyses have reported that Kinesio^®^ taping may be associated with statistically significant improvements in the total WOMAC score and physical function subscale scores in individuals with knee OA, although the pooled effect sizes are generally small-to-moderate [18, 36]. However, statistical significance does not mean clinical relevance. In this population, a 17% reduction in the WOMAC score has been identified as the MCID, yet most pooled estimates remain below this threshold [32].

The effects of Kinesio^®^ taping on function vary over time, from immediately after the implementation to the completion of the treatment process and the follow-up period. Randomized controlled trials suggests the effectiveness of taping is generally strongest during the acute phase of implementation, while this effect gradually decreases in the long term [10, 17]. This decreasing trend may be associated with the sensory adaptation that cutaneous mechanoreceptors develop over time to continuous mechanical stimulation, as supported by Kalron and Bar-Sela [41]. For this reason, current evidence suggests that the effect should be supported by treatments such as exercise or physical therapy to maintain its effectiveness [42]. Castrogiovanni et al. showed that Kinesio^®^ taping applied with exercise produced earlier and more pronounced functional improvement compared with exercise alone, with maintenance of this effect for at least 3 months [16]. The systematic review and meta-analysis by Lin et al. confirmed that physical therapy combined with Kinesio^®^ taping produced significantly greater improvements in function than physical therapy alone, and recommended applying the tape prior to exercise sessions to maximize therapeutic synergy [18]. Overall, the available findings are more consistent with Kinesio^®^ taping serving as an adjunctive tool that may prepare the individual for rehabilitation and facilitate participation in exercise, rather than as a standalone, long-term treatment.

The effects of Kinesio^®^ taping on tasks such as the Timed Up and Go (TUG) test and climbing stairs are also controversial. Although some studies reported that Kinesio^®^ taping reduces the time on these tests, others have found no significant difference [35]. Also, Ouyang et al. reported that elastic tapes like Kinesio^®^ tape are insufficient for tasks requiring vertical movement, such as climbing stairs, whereas rigid tapes show a significant effect on these tasks [43]. Similarly, Mutlu et al. reported that Kinesio^®^ taping significantly reduced time on tasks such as walking, but did not create a significant difference compared to the control group in tasks such as climbing stairs or sitting and standing [34]. Vertical tasks, such as climbing stairs, require much higher quadriceps strength and patellofemoral compression force than horizontal tasks such as walking [44]. Taken together, these findings suggest that the elastic nature of Kinesio^®^ taping may be insufficient to provide the mechanical stabilization required under such high biomechanical demands. While the sensory feedback generated by the tape appears to modulate pain and improve gait speed during low loading tasks such as walking, its contribution seems to diminish in vertical loading scenarios where patellar malalignment plays a more dominant role. This may reflect the inherently limited capacity of an elastic taping to alter joint alignment, particularly when compressive forces exceed what cutaneous input alone can compensate for.

### Muscle function outcomes

The most significant impact of knee OA on muscle structure is arthrogenic muscle inhibition, occurring secondarily to muscle atrophy, pain, and joint effusion, particularly in the periarticular muscles, especially the quadriceps femoris. Several theoretical mechanisms have been offered for how Kinesio^®^ taping may affect muscle function in this context, although direct physiological evidence specific to Kinesio^®^ taping remains limited and variable. It has been hypothesized that the lifting of the skin by the tape may increase interstitial space and thereby improve blood and lymphatic circulation, and that the stimulation of cutaneous mechanoreceptors could modulate gamma motor neuron excitability and the muscle spindle reflex [45]. Pamuk and Yucesoy demonstrated by MRI that Kinesio^®^ tape produces heterogeneous tissue deformations not only in superficial layers but also in deep fascial structures, supporting the concept of altered myofascial force transmission [31]. In contrast, several studies that have directly examined the proposed neurophysiological mechanisms have failed to demonstrate consistent physiological changes that can be specifically attributed to Kinesio^®^ taping. Mak et al. and Poon et al. have shown that Kinesio^®^ taping produces surface electromyographic (EMG) and force responses similar to sham or no taping conditions [25, 26]. Taken together, these observations indicate that the mechanisms most frequently invoked to explain muscle effects of Kinesio^®^ taping should be regarded as plausible hypotheses rather than as established physiological actions of the tape.

Some systematic reviews and meta-analyses reported that Kinesio^®^ taping did not show a significant difference in quadriceps strength or lower extremity kinetics compared to placebo or control groups [8, 46]. However, the effect of Kinesio^®^ taping on muscle strength shows variable results depending on the measurement method. One meta-analysis reported that Kinesio^®^ taping had moderately significant effects on isokinetic muscle strength, but no significant effect on isometric muscle strength measured with a handheld dynamometer [19]. Anandkumar et al. suggested that observed improvements in isokinetic quadriceps torque may be due to testing bias, as changes rarely exceeded minimal detectable change thresholds [35].

Another critical role of Kinesio^®^ taping on muscle balance in knee OA is the regulation of the agonist-antagonist relationship. The effect of Kinesio^®^ tape is different in the antagonist muscle group. Excessive co-activation in the hamstring muscle can be observed to compensate for quadriceps muscle weakness in individuals who have knee OA [47]. The literature shows that taping applied to the hamstring muscle leads to a significant decrease in muscle strength in the acute phase, but does not provide superiority compared to the control group in follow-up periods [46]. The fact that taping applied for facilitation purposes results in inhibition in the acute phase suggests that in antagonistic and highly toned muscle groups, such as the hamstrings, it may create a tone-regulating or inhibitory effect rather than a strength-enhancing effect in the acute phase.

Studies on Kinesio^®^ taping on muscle activation have mostly been conducted on healthy individuals. A study that was conducted by Mesci et al. reported that in healthy individuals with high pre-taping muscle activity, the facilitation effect was less significant, although the inhibition effect was more significant. It was suggested that in healthy individuals, the tape provides greater gains in muscles with low activity (ceiling effect theory) [48]. However, this theory was not always confirmed in individuals with knee OA. Ataş et al. reported that the implementation did not acutely alter rectus femoris activation in individuals with knee OA [17]. This result can be interpreted as suggesting that persistent reflex inhibition caused by intra-articular fluid accumulation and inflammation may attenuate the response to superficial skin stimulation. However, this explanation should be considered as a possible neurophysiological hypothesis that requires further validation through future studies, rather than as a definitive mechanism.

In conclusion, Kinesio^®^ taping should not be regarded as a direct muscle-strengthening tool. Rather, it may contribute to neuromuscular control during dynamic activities and to the regulation of agonist–antagonist tone balance. Therefore, the use of the tape alone appears insufficient, and when combined with exercise, it may have additive effects [49, 50], although the magnitude and consistency of this benefit remain uncertain.

### Proprioception outcomes

In knee OA, there is a decrease in the number and function of mechanoreceptors located in the tissues surrounding the joint (e.g., ruffini and pacini corpuscles and the golgi tendon organ), which detect mechanical stimuli [51]. This degeneration causes a significant impairment in proprioceptive accuracy in individuals with knee OA compared to healthy individuals. Proprioception tends to be impaired not only in the affected knee but also in the healthy contralateral knee. The asymptomatic proprioceptive impairment in the contralateral knee suggests that it may be influenced not only by cartilage damage but also by systemic factors such as deconditioning because of decreased physical activity or impaired sensory integration in the central nervous system [52].

Kinesio^®^ tape continuously stimulates low-threshold mechanoreceptors (e.g., ruffini and interstitial receptors), which can improve joint position sense by increasing afferent feedback to the brain [53]. However, this mechanism remains largely hypothetical, since direct neurophysiological evidence demonstrating that these cutaneous afferent changes actually occur during Kinesio^®^ taping in patients with knee OA is currently limited, and most supporting data are derived from healthy participants. Literature indicates that the proprioceptive effect of Kinesio^®^ taping is much more significant in individuals who have OA and initial sensory deficits than in healthy individuals [54]. Within this framework, Kinesio^®^ tape may be best viewed as a potential sensory aid that may transiently compensate for impaired proprioceptive input rather than as a treatment that restores joint mechanoreceptor function.

The results of a meta-analysis by Li et al. reported that Kinesio^®^ tape provided statistically significant improvements in proprioception. However, it was also noted that this effect was angle-specific. Kinesio^®^ tape significantly increased proprioceptive sensitivity at angles such as 15° and 30°, while it did not create a statistically significant difference at the 45° angle [36]. Similarly, randomized controlled trial by Cho et al. showed that the tape improved proprioception in individuals with knee OA with a high effect size (1.73–1.89) [9]. The fact that proprioceptive gain occurs at angles close to extension, such as 15° and 30°, is clinically important. These angles are the functional ranges where the knee needs stabilization the most during the load-bearing phases of gait. This may help to explain why proprioceptive gains with Kinesio^®^ taping have been reported alongside improvements in walking-related performance [43]. In conclusion, Kinesio^®^ taping may contribute to proprioceptive performance in individuals with knee OA by increasing cutaneous feedback, with the available evidence primarily supporting an effect in gait related, low-load functional aspects.

### Knee range of motion outcomes

Individuals who have knee OA were shown to have a significantly reduced total range of motion in the knee compared to healthy controls. Childs et al. reported elevated co-activation of the vastus lateralis and medial hamstrings in individuals with OA, with muscle activation duration approximately 1.5 times longer than in healthy controls, which prevents the knee from reaching either full extension or sufficient flexion, resulting in a reduced total range of motion [55]. Also, structural factors such as osteophytes, effusion, and capsular thickening on the joint surface also create a mechanical barrier at the endpoints of movement, restricting mobility [56].

In the literature, meta-analyses and randomized controlled trials report that Kinesio^®^ tape provides significantly positive changes in flexion range of motion; however, its effects on extension are controversial. The results of a meta-analysis by Aldalati et al. showed that Kinesio^®^ tape significantly increased knee flexion compared to sham taping, but did not have a statistically significant effect on extension [14]. Randomized controlled trials are consistent with this synthesis. Cho et al. reported a significant increase of 21% in pain-free active range of motion immediately following a single-session Kinesio^®^ taping [9]. Başkurt et al. observed short-term improvements in knee flexion within the Kinesio^®^ taping group [57]. Given that Kinesio^®^ taping is insufficient in correcting knee extension loss, this restriction may shift the biomechanical load centrally, leading to progressive structural damage such as meniscal extrusion and bone marrow lesions in the central compartment over time [58].

Loss of flexion is largely because of soft tissue factors such as pain and edema [8]. The edema-reducing and pain-modulating effect of Kinesio^®^ tape can reduce this soft tissue limitation in the flexion direction, allowing for an increase in range of motion. Kinesio^®^ taping is not an option to release advanced contractures or correct bony deformities in knee OA. When pain and edema limits movement, Kinesio^®^ taping may serve as an adjunctive functional facilitator that could help the patient to utilize their existing anatomical capacity. But any range-of-motion gain should be interpreted as short-term functional facilitation rather than structural change.

### Comparison with other adjunctive treatments

International guidelines recommend exercise, patient education and weight management as core interventions for knee OA, with Kinesio^®^ taping considered an adjunctive option [6, 59]. A recent network meta-analysis by Chen et al., which synthesized data from 139 randomized trials involving 12 non-pharmacological interventions, ranked the knee brace as having the highest overall probability of benefit, followed by hydrotherapy and exercise. In contrast, Kinesio^®^ taping demonstrated a lower and less consistent ranking, performing below the knee brace, exercise, and high-intensity laser therapy in several outcomes and surpassing ultrasound or short-wave diathermy only in selected comparisons rather than consistently across all outcomes [60]. Similarly, a network meta-analysis confined to nine externally applied non-pharmacological interventions placed Kinesio^®^ taping below extracorporeal shock-wave therapy, radiofrequency, and Tuina therapy on both short- and long-term VAS and WOMAC outcomes, although all included modalities were superior to sham intervention [61]. Fazli et al. randomized 56 patients with knee OA to four weeks of either Kinesio^®^ taping or a flexible neoprene knee orthosis. Both interventions similarly improved pain, WOMAC function, and quadriceps strength. Although the orthosis group showed broader improvements in proprioceptive measures, no significant between-group differences were observed at week 4 [62]. These findings suggest that continuously worn external support may provide more proprioceptive input than elastic taping. An umbrella review by Ferreira et al. classified standard exercise as having gold-level evidence for reducing pain and improving physical function in knee OA, whereas Kinesio^®^ taping, manual therapy, ultrasound, acupuncture, and interferential current were classified as having silver-level evidence [63].

Taken together, these conclusions suggest that Kinesio^®^ taping is not biomechanically equivalent to rigid bracing or to modalities with deeper tissue penetration, such as extracorporeal shock-wave therapy or radiofrequency. Its ease of compatibility with active rehabilitation nevertheless support its use as an adjunctive option, particularly when the rehabilitative goal is sensory-motor facilitation rather than mechanical correction.

### Safety and adverse effects

Kinesio^®^ taping is generally considered a safe and well tolerated intervention. Systematic reviews have reported that Kinesio^®^ taping is not associated with serious adverse outcomes in individuals with knee OA [36, 38]. Reported adverse events in knee OA studies have mainly involved minor and self-limiting skin reactions such as transient erythema, mild itching, contact dermatitis, and skin irritation at the tape edges. These are rare, resolve after the tape is removed, and do not lead to discontinuation of treatment [64, 65]. Allergic reactions to the adhesive have been reported in a small proportion of patients and constitute the most common reason for discontinuing treatment [64]. Therefore, it is recommended to screen the application site for sensitivity, new skin injuries, or open wounds prior to treatment.

## Strengths and limitations

This narrative review has several strengths. First, it integrates evidence from systematic reviews, meta-analyses, randomized controlled trials, and observational studies into a mechanism-driven synthesis that links the biomechanical, neurophysiological, and clinical outcomes of Kinesio^®^ taping in knee OA. Second, the review evaluates pain, function, muscle activation, proprioception, and range of motion separately, allowing the contexts in which Kinesio^®^ taping does and does not provide meaningful benefit to be identified. Third, throughout the review, statistical significance is consistently distinguished from clinical significance, and findings are interpreted with reference to the MCID wherever the data permit.

Several limitations should also be acknowledged. As this is a narrative review, it is not necessary to conduct a risk of bias assessment or GRADE evaluation. Therefore reflects an interpretive rather than a quantitative weighting of evidence. The literature exhibits substantial heterogeneity in taping technique, tension level, application duration, comparator, and outcome measurement, which limits the generalizability of any single conclusion.

## Conclusion

Current evidence does not support routine recommendation of Kinesio^®^ taping as a stand-alone intervention for knee OA because clinically meaningful effects beyond placebo or sham conditions are inconsistent. Kinesio^®^ taping may be used together with exercise and patient education in selected patients, mainly to reduce symptoms, enhance sensory feedback, and support participation in low-load functional activities. It should not be viewed as a mechanical corrector, a disease-modifying therapy, or a replacement for standard OA management. Its clinical use should be guided by the patient’s response, treatment goals, OA severity, and evidence of meaningful benefit on reassessment. Future randomized controlled trials in knee OA should standardize the taping protocol and report it in sufficient detail to permit replication. Double-blind, sham-controlled trials with adequately sample sizes and follow-up periods of at least 6–12 months are needed to determine translate into clinically meaningful long-term outcomes. The mechanistic basis of Kinesio^®^ taping in knee OA should be examined directly in patients with knee OA, using electromyography, ultrasonography, and quantitative sensory testing.

## Declarations

### Disclaimer

This manuscript has not been published elsewhere, in whole or in part, nor copied from any other source.

### Data availability

Data sharing is not applicable to this article as no new data were created or analyzed in this review.

## References

[1] Wieland HA, Michaelis M, Kirschbaum BJ, Rudolphi KA (2005) Osteoarthritis—an untreatable disease? Nat Rev Drug Discov 4:331–344. 10.1038/nrd1693 15803196 10.1038/nrd1693

[2] Hunter DJ, Bierma-Zeinstra S (2019) Osteoarthritis. Lancet 393:1745–1759. 10.1016/s0140-6736(19)30417-9 31034380 10.1016/S0140-6736(19)30417-9

[3] Langworthy M, Dasa V, Spitzer AI (2024) Knee osteoarthritis: disease burden, available treatments, and emerging options. Ther Adv Musculoskelet Dis 16:1759720x241273009. 10.1177/1759720x241273009 39290780 10.1177/1759720X241273009 PMC11406648

[4] Steinmetz JD, Culbreth GT, Haile LM, Rafferty Q, Lo J, Fukutaki KG, Cruz JA, Smith AE, Vollset SE, Brooks PM et al (2023) Global, regional, and national burden of osteoarthritis, 1990–2020 and projections to 2050: a systematic analysis for the Global Burden of Disease Study 2021. Lancet Rheumatol 5:e508–e522. 10.1016/S2665-9913(23)00163-7 37675071 10.1016/S2665-9913(23)00163-7 PMC10477960

[5] Zhu S, Qu W, He C (2024) Evaluation and management of knee osteoarthritis. J Evid Based Med 17:675–687. 10.1111/jebm.12627 38963824 10.1111/jebm.12627

[6] McColm S, Ackerman P, Graham V, Hamilton DF (2025) Consistency of advice for the conservative management of knee osteoarthritis across international clinical practice guidelines. Bone Jt Open 6:1358–1370. 10.1302/2633-1462.611.Bjo-2024-0153.R1 41183559 10.1302/2633-1462.611.BJO-2024-0153.R1 PMC12582647

[7] Kase K, Wallis J, Kase T (2003) Clinical therapeutic applications of the kinesio taping method, 2nd edn. Ken Ikai Co Ltd, Tokyo

[8] Lu Z, Li X, Chen R, Guo C (2018) Kinesio taping improves pain and function in patients with knee osteoarthritis: a meta-analysis of randomized controlled trials. Int J Surg 59:27–35. 10.1016/j.ijsu.2018.09.015 30273684 10.1016/j.ijsu.2018.09.015

[9] Cho H-y, Kim E-H, Kim J, Yoon YW (2015) Kinesio taping improves pain, range of motion, and proprioception in older patients with knee osteoarthritis: a randomized controlled trial. Am J Phys Med Rehabil 94:192–200. 10.1097/PHM.0000000000000148 25706053 10.1097/PHM.0000000000000148

[10] Wageck B, Nunes GS, Bohlen NB, Santos GM, de Noronha M (2016) Kinesio taping does not improve the symptoms or function of older people with knee osteoarthritis: a randomised trial. J Physiother 62:153–158. 10.1016/j.jphys.2016.05.012 27320828 10.1016/j.jphys.2016.05.012

[11] Kocyigit F, Turkmen MB, Acar M, Guldane N, Kose T, Kuyucu E, Erdil M (2015) Kinesio taping or sham taping in knee osteoarthritis? A randomized, double-blind, sham-controlled trial. Complement Ther Clin Pract 21:262–267. 10.1016/j.ctcp.2015.10.001 26573453 10.1016/j.ctcp.2015.10.001

[12] Lemos TV, de Souza Júnior JR, Dos Santos MGR, Rosa MMN, da Silva LGC, Matheus JPC (2018) Kinesio taping effects with different directions and tensions on strength and range of movement of the knee: a randomized controlled trial. Braz J Phys Ther 22:283–290 29728298 10.1016/j.bjpt.2018.04.001 PMC6095100

[13] Morris D, Jones D, Ryan H, Ryan C (2013) The clinical effects of Kinesio^®^ tex taping: a systematic review. Physiother Theory Pract 29:259–270. 10.3109/09593985.2012.731675 23088702 10.3109/09593985.2012.731675

[14] Aldalati AY, Hussein AM, Hammadeh BM, Alrabadi B, Albliwi M, Abuassi M (2025) Effectiveness of kinesio taping without physical therapy for knee osteoarthritis: a systematic review and meta-analysis of randomized controlled trials. Rheumatol Int 45:99. 10.1007/s00296-025-05853-z 40232317 10.1007/s00296-025-05853-z

[15] Gasparyan AY, Ayvazyan L, Blackmore H, Kitas GD (2011) Writing a narrative biomedical review: considerations for authors, peer reviewers, and editors. Rheumatol Int 31:1409–1417. 10.1007/s00296-011-1999-3 21800117 10.1007/s00296-011-1999-3

[16] Castrogiovanni P, Di Giunta A, Guglielmino C, Roggio F, Romeo D, Fidone F, Imbesi R, Loreto C, Castorina S, Musumeci G (2016) The effects of exercise and kinesio tape on physical limitations in patients with knee osteoarthritis. J Funct Morphol Kinesiol 1:355–368. 10.3390/jfmk1040355

[17] Ataş A, Abit Kocaman A, Karaca ŞB, Kasikci Çavdar M (2024) Acute effect of kinesiology taping on muscle activation, functionality and proprioception in patients with knee osteoarthritis: a randomized controlled trial. Percept Mot Skills 131:446–468. 10.1177/00315125231222816 38134448 10.1177/00315125231222816

[18] Lin C-H, Lee M, Lu K-Y, Chang C-H, Huang S-S, Chen C-M (2020) Comparative effects of combined physical therapy with kinesio taping and physical therapy in patients with knee osteoarthritis: a systematic review and meta-analysis. Clin Rehabil 34:1014–1027. 10.1177/0269215520928398 32597199 10.1177/0269215520928398

[19] Mao H-Y, Hu M-T, Yen Y-Y, Lan S-J, Lee S-D (2021) Kinesio taping relieves pain and improves isokinetic not isometric muscle strength in patients with knee osteoarthritis—a systematic review and meta-analysis. Int J Environ Res Public Health 18:10440. 10.3390/ijerph181910440 34639740 10.3390/ijerph181910440 PMC8507801

[20] Pinheiro YT, Barbosa GM, Fialho HRF, Silva CAM, de Oliveira Anunciação J, de Almeida Silva HJ, de Souza MC, de Almeida Lins CA (2020) Does tension applied in kinesio taping affect pain or function in older women with knee osteoarthritis? A randomised controlled trial. BMJ Open 10:e041121. 10.1136/bmjopen-2020-041121 33328259 10.1136/bmjopen-2020-041121 PMC7745684

[21] Mahmoud WS (2024) Effect of kinesio taping and exercise on functional impairment in patients with different degrees of knee osteoarthritis. Isokinet Exerc Sci 32:133–143. 10.3233/Ies-230028

[22] Yuksel E, Unver B, Karatosun V (2022) Comparison of kinesio taping and cold therapy in patients with total knee arthroplasty: a randomized controlled trial. Clin Rehabil 36:359–368. 10.1177/02692155211049152 34672833 10.1177/02692155211049152

[23] Ataabadi PA, Abbasi A, Shojaatian M, Letafatkar A, Svoboda Z, Rossettini G (2022) The effects of facilitatory and inhibitory kinesiotaping of Vastus Medialis on the activation and fatigue of superficial quadriceps muscles. Sci Rep 12:13451. 10.1038/s41598-022-17849-x 35927291 10.1038/s41598-022-17849-x PMC9352761

[24] Choi IR, Lee JH (2018) Effect of kinesiology tape application direction on quadriceps strength. Med (Baltim) 97:e11038. 10.1097/MD.0000000000011038 10.1097/MD.0000000000011038 PMC6023644 29901599

[25] Mak DN-T, Au IP-H, Chan M, Chan ZY-S, An WW, Zhang JH, Draper D, Cheung RT-H (2019) Placebo effect of facilitatory kinesio tape on muscle activity and muscle strength. Physiother Theory Pract 35:157–162. 10.1080/09593985.2018.1441936 29461139 10.1080/09593985.2018.1441936

[26] Poon KY, Li S, Roper M, Wong M, Wong O, Cheung R (2015) Kinesiology tape does not facilitate muscle performance: a deceptive controlled trial. Man Ther 20:130–133. 10.1016/j.math.2014.07.013 25150913 10.1016/j.math.2014.07.013

[27] Ye W, Jia C, Jiang J, Liang Q, He C (2020) Effectiveness of elastic taping in patients with knee osteoarthritis: a systematic review and meta-analysis. Am J Phys Med Rehabil 99:495–503. 10.1097/PHM.0000000000001361 31851010 10.1097/PHM.0000000000001361

[28] Wu W-T, Hong C-Z, Chou L-W (2015) The kinesio taping method for myofascial pain control. Evid Based Complement Alternat Med 2015:950519. 10.1155/2015/950519 26185522 10.1155/2015/950519 PMC4491400

[29] Tang Sa, Zhang C, Oo WM, Fu K, Risberg MA, Bierma-Zeinstra SM, Neogi T, Atukorala I, Malfait A-M, Ding C, Hunter DJ (2025) Osteoarthritis. Nat Rev Dis Primers 11:10. 10.1038/s41572-025-00594-6 39948092 10.1038/s41572-025-00594-6

[30] Melzack R, Wall PD (1965) Pain mechanisms: a new theory: a gate control system modulates sensory input from the skin before it evokes pain perception and response. Science 150:971–979. 10.1126/science.150.3699.971 5320816 10.1126/science.150.3699.971

[31] Pamuk U, Yucesoy CA (2015) MRI analyses show that kinesio taping affects much more than just the targeted superficial tissues and causes heterogeneous deformations within the whole limb. J Biomech 48:4262–4270. 10.1016/j.jbiomech.2015.10.036 26556717 10.1016/j.jbiomech.2015.10.036

[32] Heddon S, Saulnier N, Mercado J, Shalmiyev M, Berteau J-P (2021) Systematic review shows no strong evidence regarding the use of elastic taping for pain improvement in patients with primary knee osteoarthritis. Med (Baltim) 100:e25382. 10.1097/MD.0000000000025382 10.1097/MD.0000000000025382 PMC8021313 33787644

[33] Parreira PCS, Costa LCM, Junior LCH, Lopes AD, Costa LOP (2014) Current evidence does not support the use of kinesio taping in clinical practice: a systematic review. J Physiother 60:31–39. 10.1016/j.jphys.2013.12.008 24856938 10.1016/j.jphys.2013.12.008

[34] Mutlu EK, Mustafaoglu R, Birinci T, Ozdincler AR (2017) Does kinesio taping of the knee improve pain and functionality in patients with knee osteoarthritis? A randomized controlled clinical trial. Am J Phys Med Rehabil 96:25–33. 10.1097/PHM.0000000000000520 27149590 10.1097/PHM.0000000000000520

[35] Anandkumar S, Sudarshan S, Nagpal P (2014) Efficacy of kinesio taping on isokinetic quadriceps torque in knee osteoarthritis: a double blinded randomized controlled study. Physiother Theory Pract 30:375–383. 10.3109/09593985.2014.896963 24617598 10.3109/09593985.2014.896963

[36] Li X, Zhou X, Liu H, Chen N, Liang J, Yang X, Zhao G, Song Y, Du Q (2018) Effects of elastic therapeutic taping on knee osteoarthritis: a systematic review and meta-analysis. Aging Dis 9:296. 10.14336/AD.2017.0309 29896418 10.14336/AD.2017.0309 PMC5963350

[37] Mostafavifar M, Wertz J, Borchers J (2012) A systematic review of the effectiveness of kinesio taping for musculoskeletal injury. Phys Sportsmed 40:33–40. 10.3810/psm.2012.11.1986 23306413 10.3810/psm.2012.11.1986

[38] Wu H, Yao R, Wu J, Wen G, Wang Y (2022) Does kinesio taping plus exercise improve pain and function in patients with knee osteoarthritis? A systematic review and meta-analysis of randomized controlled trials. Front Physiol 13:961264. 10.3389/fphys.2022.961264 36160871 10.3389/fphys.2022.961264 PMC9500481

[39] Huang KH, Hsieh RL, Lee WC (2015) Pain, physical function, and health in patients with knee osteoarthritis. Rehabil Nurs. 10.1002/rnj.234 10.1002/rnj.234 26391619

[40] Donec V, Kubilius R (2020) The effectiveness of kinesio taping^®^ for mobility and functioning improvement in knee osteoarthritis: a randomized, double-blind, controlled trial. Clin Rehabil 34:877–889. 10.1177/0269215520916859 32372651 10.1177/0269215520916859 PMC7376619

[41] Kalron A, Bar-Sela S (2013) A systematic review of the effectiveness of kinesio taping—fact or fashion. Eur J Phys Rehabil Med 49:699–709 23558699

[42] Altaş EU, Günay Uçurum S, Ozer Kaya D (2021) Acute effect of kinesiology taping on muscle strength, tissue temperature, balance, and mobility in female patients with osteoarthritis of the knee. Somatosens Mot Res 38:48–53. 10.1080/08990220.2020.1840347 33115305 10.1080/08990220.2020.1840347

[43] Ouyang J-H, Chang K-H, Hsu W-Y, Cho Y-T, Liou T-H, Lin Y-N (2018) Non-elastic taping, but not elastic taping, provides benefits for patients with knee osteoarthritis: systemic review and meta-analysis. Clin Rehabil 32:3–17. 10.1177/0269215517717307 28660785 10.1177/0269215517717307

[44] Taheri P, Vahdatpour B, Asl MM, Ramezanian H (2017) Effects of taping on pain and functional outcome of patients with knee osteoarthritis: a pilot randomized single-blind clinical trial. Adv Biomed Res 6:139. 10.4103/2277-9175.218031 29279837 10.4103/2277-9175.218031 PMC5698981

[45] Konishi Y (2013) Tactile stimulation with kinesiology tape alleviates muscle weakness attributable to attenuation of Ia afferents. J Sci Med Sport 16:45–48. 10.1016/j.jsams.2012.04.007 22682093 10.1016/j.jsams.2012.04.007

[46] Karabicak GO, Ozunlu Pekyavas N, Baltaci G, Karacam Z (2023) Does kinesio taping have an effect on kinetics and kinematics after lower limb musculoskeletal injuries? Systematic review and meta-analysis. Disabil Rehabil 45:3639–3648. 10.1080/09638288.2022.2134467 36269093 10.1080/09638288.2022.2134467

[47] Murphy MT, Wang N, Felson DT, Nevitt MC, Lewis CE, Frey-Law L, Guermazi A, Segal NA (2022) Association between hamstring coactivation during isokinetic quadriceps strength testing and knee cartilage worsening over 24 months. Osteoarthritis Cartilage 30:823–831. 10.1016/j.joca.2022.03.002 35307535 10.1016/j.joca.2022.03.002 PMC9450915

[48] Mesci N, Yorulmaz E, Külcü DG, Baydili KN, Mesci E (2024) Can the effects of muscle techniques in kinesiotaping be demonstrated objectively in female healthy individuals? An electrophysiological study. Turk J Phys Med Rehabil 71:427–434. 10.5606/tftrd.2024.16138 41717517 10.5606/tftrd.2024.16138 PMC12914252

[49] Danazumi MS, Ibrahim SU, Yakasai AM, Dermody G, Bello B, Kaka B (2021) A comparison between the effect of combined chain exercises plus kinesio taping with combined chain exercises alone in knee osteoarthritis: a randomized clinical trial. Am J Phys Med Rehabil 100:1070–1077. 10.1097/PHM.0000000000001705 33496439 10.1097/PHM.0000000000001705

[50] Nayanti AP, Prabowo T, Sari DM (2020) The effects of kinesio taping and quadriceps muscle strengthening exercise on quadriceps muscle strength and functional status in knee osteoarthritis. J Med Health. 10.28932/jmh.v2i5.1555

[51] Salamanna F, Caravelli S, Marchese L, Carniato M, Vocale E, Gardini G, Puccetti G, Mosca M, Giavaresi G (2023) Proprioception and mechanoreceptors in osteoarthritis: a systematic literature review. J Clin Med 12:6623. 10.3390/jcm12206623 37892761 10.3390/jcm12206623 PMC10607296

[52] Knoop J, Steultjens M, Van der Leeden M, Van der Esch M, Thorstensson C, Roorda L, Lems W, Dekker J (2011) Proprioception in knee osteoarthritis: a narrative review. Osteoarthritis Cartilage 19:381–388. 10.1016/j.joca.2011.01.003 21251988 10.1016/j.joca.2011.01.003

[53] Halseth T, McChesney JW, DeBeliso M, Vaughn R, Lien J (2004) The effects of kinesio™ taping on proprioception at the ankle. J Sports Sci Med 3:1 24497814 PMC3896108

[54] Simon J, Garcia W, Docherty CL (2014) The effect of kinesio tape on force sense in people with functional ankle instability. Clin J Sport Med 24:289–294. 10.1097/JSM.0000000000000030 24184853 10.1097/JSM.0000000000000030

[55] Childs JD, Sparto PJ, Fitzgerald GK, Bizzini M, Irrgang JJ (2004) Alterations in lower extremity movement and muscle activation patterns in individuals with knee osteoarthritis. Clin Biomech (Bristol Avon) 19:44–49. 10.1016/j.clinbiomech.2003.08.007 10.1016/j.clinbiomech.2003.08.007 14659929

[56] Györy AN, Chao E, Stauffer RN (1976) Functional evaluation of normal and pathologic knees during gait. Arch Phys Med Rehabil 57:571–577 999482

[57] Başkurt Z, Ercan S, Parpucu Tİ, Başkurt F, Ünal M (2017) Short term effects of kinesiotape application in patients with knee osteoarthritis. Turk J Sports Med. 10.5152/tjsm.2017.082

[58] Chan Chun Kong D, Conaghan PG, Campbell TM (2025) Baseline loss of knee extension is associated with regional MRI progression in knee osteoarthritis: a retrospective longitudinal cohort study—data from the osteoarthritis initiative. Rheumatol Int 45:1–10. 10.1007/s00296-025-06008-w 10.1007/s00296-025-06008-w 41128850

[59] Mehta SP, Tate J, Donachy B, White T, Keinath C (2026) A systematic review of clinical practice guidelines for physical therapy management of hip and knee osteoarthritis. Crit Rev Phys Rehabil Med. 10.1615/CritRevPhysRehabilMed.2025059275

[60] Chen X, Fan Y, Tu H, Luo Y (2025) Clinical efficacy of different therapeutic options for knee osteoarthritis: a network meta-analysis based on randomized clinical trials. PLoS ONE 20:e0324864. 10.1371/journal.pone.0324864 40531843 10.1371/journal.pone.0324864 PMC12176148

[61] Wang Z, Xu H, Wang Z, Zhou H, Diao J, Zhang L, Wang Y, Li M, Zhou Y (2023) Effects of externally-applied, non-pharmacological Interventions on short-and long-term symptoms and inflammatory cytokine levels in patients with knee osteoarthritis: a systematic review and network meta-analysis. Front Immunol 14:1309751. 10.3389/fimmu.2023.1309751 38155966 10.3389/fimmu.2023.1309751 PMC10752972

[62] Fazli F, Farsi A, Takamjani IE, Sohani SM, Yousefi N, Azadinia F (2023) Effect of knee orthosis and kinesio taping on clinical and neuromuscular outcomes in patients with knee osteoarthritis: a randomized clinical trial. Arch Bone Jt Surg 11:625. 10.22038/ABJS.2023.72208.3362 37873530 10.22038/ABJS.2023.72208.3362 PMC10590486

[63] Ferreira RM, Duarte JA, Gonçalves RS (2018) Non-pharmacological and non-surgical interventions to manage patients with knee osteoarthritis: an umbrella review. Acta Reumatol Port 43:182–200 30414367

[64] de Almeida Alcantara DA, Dos Santos FNA, de Almeida Ferreira JJ, de Noronha M, de Andrade PR (2024) The effect of kinesiotaping on edema: a systematic review and meta-analysis. Musculoskelet Sci Pract 74:103168. 10.1016/j.msksp.2024.103168 39213979 10.1016/j.msksp.2024.103168

[65] Banerjee G, Johnson MI (2020) Should kinesiology taping be used to manage pain in musculoskeletal disorders? An evidence synthesis from systematic reviews. Physiother J Indian Assoc Physiother 14:17–25. 10.4103/PJIAP.PJIAP_31_19

